# A model for self-organization of sensorimotor function: spinal interneuronal integration

**DOI:** 10.1152/jn.00054.2022

**Published:** 2022-04-27

**Authors:** Jonas M. D. Enander, Gerald E. Loeb, Henrik Jörntell

**Affiliations:** ^1^Department of Experimental Medical Science, Faculty of Medicine, Lund University, Lund, Sweden; ^2^Department of Biomedical Engineering, Viterbi School of Engineering, University of Southern California, Los Angeles, California

**Keywords:** extrafusal muscle, interneurons, intrafusal muscle, neuron model, spinal development

## Abstract

Control of musculoskeletal systems depends on integration of voluntary commands and somatosensory feedback in the complex neural circuits of the spinal cord. It has been suggested that the various connectivity patterns that have been identified experimentally may result from the many transcriptional types that have been observed in spinal interneurons. We ask instead whether the muscle-specific details of observed connectivity patterns can arise as a consequence of Hebbian adaptation during early development, rather than being genetically ordained. We constructed an anatomically simplified model musculoskeletal system with realistic muscles and sensors and connected it to a recurrent, random neuronal network consisting of both excitatory and inhibitory neurons endowed with Hebbian learning rules. We then generated a wide set of randomized muscle twitches typical of those described during fetal development and allowed the network to learn. Multiple simulations consistently resulted in diverse and stable patterns of activity and connectivity that included subsets of the interneurons that were similar to “archetypical” interneurons described in the literature. We also found that such learning led to an increased degree of cooperativity between interneurons when performing larger limb movements on which it had not been trained. Hebbian learning gives rise to rich sets of diverse interneurons whose connectivity reflects the mechanical properties of the system. At least some of the transcriptomic diversity may reflect the effects of this process rather than the cause of the connectivity. Such a learning process seems better suited to respond to the musculoskeletal mutations that underlie the evolution of new species.

**NEW & NOTEWORTHY** We present a model of a self-organizing early spinal cord circuitry, which is attached to a biologically realistic sensorized musculoskeletal system. Without any a priori-defined connectivity or organization, learning induced by spontaneous, fetal-like motor activity results in the emergence of a well-functioning spinal interneuronal circuit whose connectivity patterns resemble in many respects those observed in the adult mammalian spinal cord. Hence, our result questions the importance of genetically controlled wiring for spinal cord function.

## Graphical abstract

**Figure:**
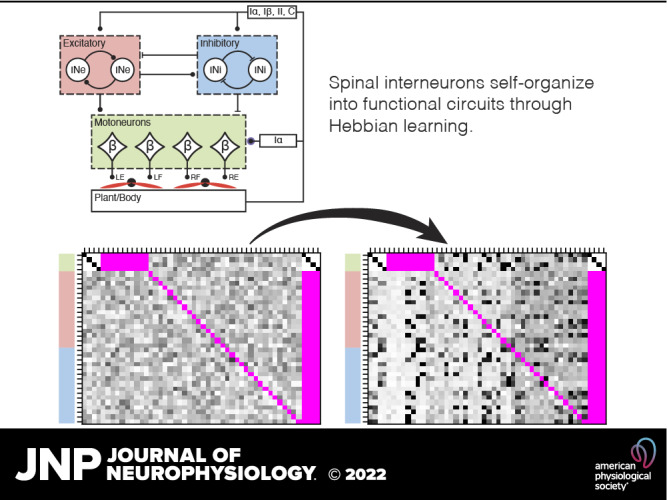

## INTRODUCTION

The organization and function of the sensorimotor interneurons of the mammalian spinal cord remain contentious after over a century of experimental characterization. They were first invoked to account for the variety of clinically observed reflexes (1, 2). Then they were interpreted as biological analogs of the servocontrollers used to stabilize position, force, or stiffness of electric motors (3, 4). The various interneurons whose inputs and outputs have been characterized electrophysiologically have been organized into types based on the nature of the first discovered or most prominent aspects of their connectivity (5–7), but their complete patterns of connectivity are quite complex and inconsistent with conventional servocontrol (8, 9). The aggregate set of interneurons has been modeled as a programmable multi-input-multi-output regulator whose design might be understood as the embodiment of a form of optimal control called linear quadratic regulator design (10, 11), but it seems unlikely that the brain could compute the myriad weights required to achieve optimal control via such circuitry (12, 13).

Most of the descending motor command signals project to the spinal interneurons rather than directly to the motoneurons, particularly for large limb muscles (14, 15). Models consisting of the classically identified interneuronal types and their proprioceptive feedback are able to generate much of the phasic muscle activation observed in learned, complex limb movements (16–18), but it is difficult to obtain recordings from identified types to validate such models (19–22). Further complexity is introduced by the plasticity of the spinal circuitry, which is readily observable as heterogeneous clinical spasticity following cerebral strokes and spinal cord injuries that interfere with the normal descending control of these interneurons (23, 24).

The spinal circuitry provides the substrate that enables the brain to achieve functional control of the complex musculoskeletal system; presumably its details matter. We are testing the hypothesis that the details of this circuitry come to reflect the mechanics of the musculoskeletal system as a result of sensorimotor experience starting at the earliest stages of embryological development (i.e., nurture), rather than genetically preordained differentiation of specific interneurons and their connectivity (i.e., nature). Such an adaptive developmental process would complement the mechanics of the musculoskeletal linkages, which are subject to ontological variability and phylogenetic evolution. Developmental mechanisms that can respond to such mechanical vicissitudes would provide a more flexible scheme than requiring simultaneous and complementary mutations of both mesenchymal (musculoskeletal) and ectodermal (neural) cell lines (25, 26).

This paper is the second of a series based on modeling development and learning of realistic neurons operating a simplified musculoskeletal system in which an extensor and flexor muscle pair in each of two limbs can produce single dimensional movement. In the first paper, we demonstrated that spontaneous, random twitches of the individual muscles such as observed during fetal development could account for the mature pattern of muscle-specific connectivity observed in the Ia-β motoneuron (βMN) synapses (27). In this paper, we took that learned pattern as a starting condition and extended Hebbian learning to initially undifferentiated sets of excitatory and inhibitory interneurons, again using the spontaneous muscle twitches as the activity generating source rather than a ready-made rhythm generating circuit. Our hypothesis was that simple Hebbian rules for adjusting synaptic weights can result in the formation of circuits that meet the following requirements for a spinal cord that would facilitate further development of learned motor behaviors:

- After randomizing the initial weights of an all-to-all pattern of connectivity, the circuitry should invariably converge to a stable and reasonably steady state.
- The observed patterns of mature connectivity can be heterogeneous, consistent with behavioral heterogeneity of biological organisms (28), but all should demonstrate increased coherence of their own activity and the resultant limb kinematics compared with the initial state.
- No interneurons should have input or output weights that are saturated at unphysiologically high values.
- Each functional interneuron should have at least a few input and output weights that are not saturated at negligible values, but some interneurons may fail to find a functional role and be marked for apoptosis (29).

The aforementioned requirements are necessary but not necessarily sufficient to provide a functional substrate for learned sensorimotor behavior. Such behaviors arise from corticospinal projections to the spinal circuitry that occur in later stages of embryological development (30) and are associated with the emergence of new patterns of observed musculoskeletal activity (31). These will be the subject of follow-on research using this model system.

## METHODS

### Model Design and Musculoskeletal Mechanics

**Figure 1.**
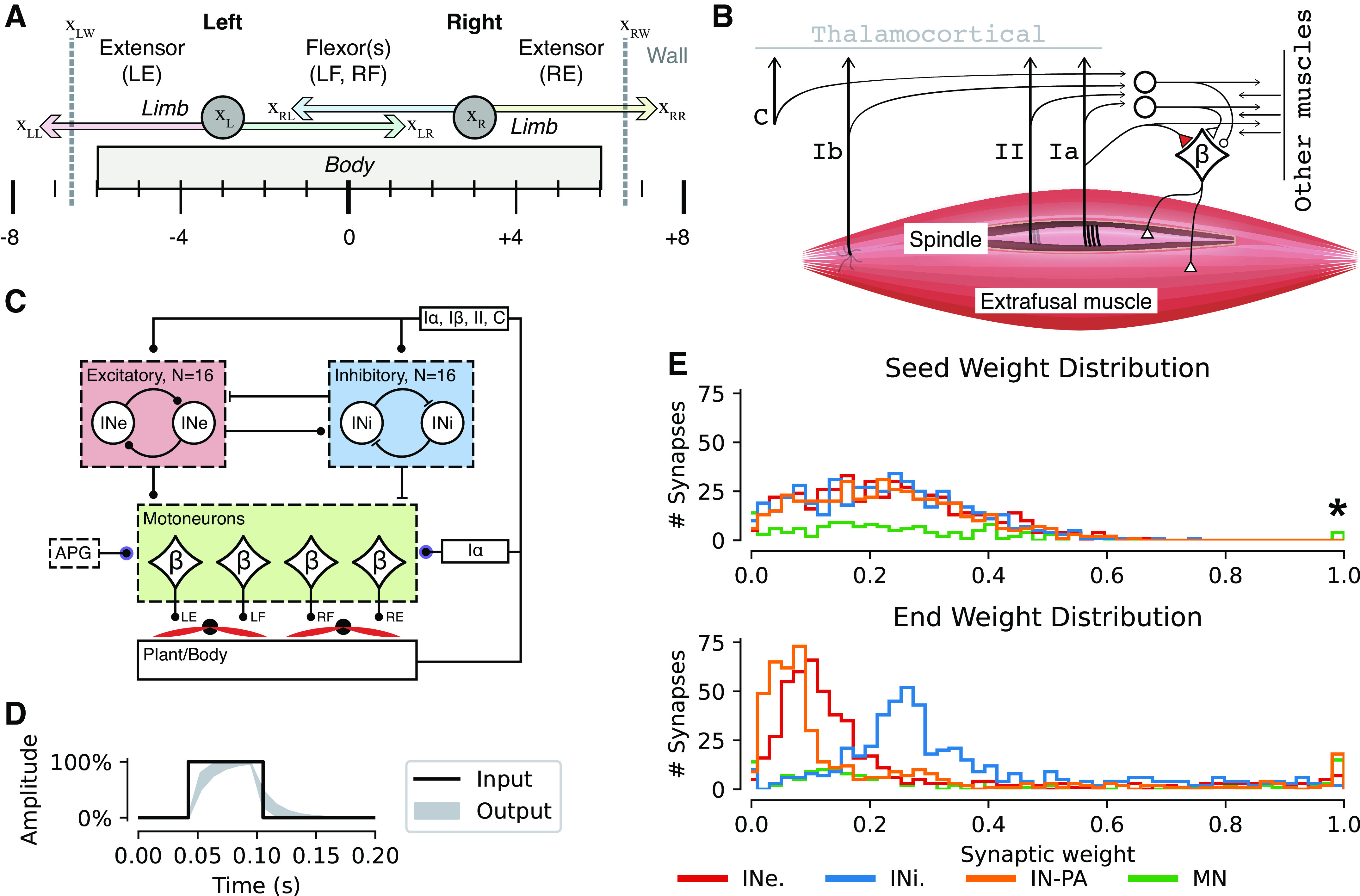
Anatomy, muscle, and network properties of the Oropod model system. *A*: the Oropod anatomy with body, limbs, and corresponding muscles: left extensor (LE), left flexor (LF), right flexor (RF), and right extensor (RE). Left and right walls of the Oropod world are indicated with gray dashed vertical lines, and the absolute global positions are indicated below the body; position terms used in *Eqs. 1–5* are relative *x*_LL_. The limbs’ anatomical limits are ±4 units, as shown. *B*: proprioceptive receptors of the Oropod muscle. In this paper we focus on the spinal circuitry, leaving the thalamocortical connections out and thus depicted in gray. *C*: illustration of the neural network design of the Oropod. The Ia afferents from each muscle are the only sensors that synapse directly onto the β motoneurons (MNs) controlling the four muscles. The homonymous Ia-βMN synapse is frozen with weight set to 100% according to our previous paper (27). All somatosensors synapse onto all interneurons. There are two sets of 16 interneurons: excitatory (INe) indicated by the red box and inhibitory (INi) indicated by the blue box. All 32 interneurons synapse onto each other (but not on themselves); all interneurons also synapse onto the four βMNs. Finally, there are four special synapses onto the βMNs from the activity pattern generator (APG), explained in detail in the methods section. *D*: example of a twitch APG with 100% amplitude and 50 ms duration; the black line shows the internal square pulse into the APG and the light gray area shows the output range when fed through the one-dimensional Kalman filter with the gain range 0.5–0.8; the output that is injected into the βMN synapse. *E*: example distribution of synaptic weights across all synapses of the neural network, before (seed) and after (end) training. All seed weights were generated from an only-positive normal distribution with mean = 0.2 and SD = 0.16. All the synaptic weights on the motoneurons, the synaptic weights of the interneuronal synapses partitioned by excitatory and inhibitory, and the synaptic weights of the primary afferents onto the interneurons are shown separately (refer to legend). The four 100% motoneuronal synaptic seed weights indicated with a star are the four homonymous Ia synapses that evolved during the earlier phase of Hebbian learning described by Enander et al. (27), which were fixed and not subject to further adaptation during the developmental phases modeled here.

The model system, or model organism, is depicted schematically in Fig. 1*A*. This is the same model organism as in our previous publication (27). The organism, called an Oropod, lives in a world bounded by walls with which it can interact. The Oropod is designed to be akin to a one-dimensional cephalopod, with two tentacle-like limbs that can be moved (up to ±4 units along the *x*-axis) so as to contact each other and to shift the Oropod’s position by pushing against the world boundary (i.e., the walls).

Each one-dimensional limb is operated by an antagonistic pair of muscles that are unidirectional muscle force-generators (springs that can pull but not push). Extensor muscles pull the limbs outward relative to the center of the Oropod body and flexor muscles pull the limbs inward and toward each other, resulting in a total of four muscles. The equations describing the physical properties of the world can be seen in *Eqs. 1–5*, where *B* denotes the viscosity; *x* is position relative to the center of the body [ranging from the maximal possible limb extension left (negative) and right (positive)]; *A* is muscle activation; *D* is damping due to viscosity; and *L*_max_ is maximal muscle length. Subscripts L and R denote left and right respectively, and subscripts LW and RW denote left and right wall respectively. Refer to Table 1 for a complete list of all variables used in this article.

(*1*) $$B\dot{x}=F_{\text{L}}=-A_{\text{LE}}x_{\text{L}}+A_{\text{LF}}\left(x_{\text{LR}}-x_{\text{L}}\right)-{\dot{x}}_{\text{L}}D-F_{\text{C}}\text{, (force left limb),}$$

(*2*) $$B\dot{x}=F_{\text{R}}=-A_{\text{RF}}\left(x_{\text{R}}-x_{\text{RL}}\right)+A_{\text{RE}}\left(x_{\text{RR}}-x_{\text{R}}\right)-{\dot{x}}_{\text{R}}D-F_{\text{C}}\text{, (force right limb),}$$

(*3*) $$F_{\text{LW}}=A_{\text{LE}}\left(L_{\text{max}}-x_{\text{LW}}-x_{\text{L}}\right)-A_{\text{LF}}(x_{\text{LR}}-x_{\text{L}}),\text{(force left wall, valid for positive forces),}$$

(*4*) $$F_{\text{RW}}=-A_{\text{RE}}\left(L_{\text{max}}-x_{\text{RW}}-x_{\text{R}}\right)+A_{\text{RF}}(x_{\text{RR}}-x_{\text{R}})\text{,}\text{ (force right wall, valid for positive forces),}$$

(*5*) FBody=FLW−FRW−x˙D, (force body valid for positive FLW and negative FRW)

**Table 1.** Parameter definitions

Parameter	Symbol	Value or range
Position along the *x*-axis relative a zero-point at the far left	*x*	−inf to inf
Damping	*D*	2.0
Muscle length	*L*	0 to 1
Muscle activation	*A*	0 to 1
Neuronal activation (membrane potential). Superscript plus denotes the positive part of the range.	*P*	−1 to 1
Sign matrix denoting excitatory or inhibitory synapses	*S*	+1 or −1
Synaptic weight. Superscript plus denotes the positive part of the range, which is the functional part of the range.	*w*	−1 to 1
Learning signal	*l*	−1 to 1
Compensation factor	*c*	0 to 1
Learning rate	η	0 to 0.001
Kalman gain for activity	*K*_A_	0.3
Kalman gain for learning signal	*K*_L_	0.001
Kalman gain for mean activity	*K*_M_	4.0*e*-5
Kalman gain for cutaneous sensor	*K*_C_	0.054
Filter function for synaptic activity. High-pass (0.05 Hz threshold) for EPSP and low-pass (0.2 Hz threshold) for IPSP.	φ_L_	Function
One-dimensional Kalman filter	φ_K_	Function
Decay function with decay constant of 0.985	φ_τ_	Function
Initial scalar for controlling decay of force during collision	τ	3.0

Similar to the biological world, in the Oropod model system the force output of a muscle is a function of both its neural activation and its kinematic condition, which gives rise to “preflex” responses to perturbations (32). The Oropod muscles incorporate the well-known “springlike” property of muscles operating on the ascending limb of their force-length curve. The spring curves have a mid-range overlap so that the organism can control “stiffness” by using different levels of co-contraction of antagonists (33). Similar to an aquatic organism, the Oropod has limb and body dynamics dominated by velocity and muscle dynamics dominated by length (limb position), whereas real terrestrial organisms have limb dynamics dominated by acceleration and muscle dynamics dominated by length and velocity (34). An in-depth discussion of the properties and design choices of the Oropod can be found in the companion paper (27).

### Somatosensation

Each Oropod muscle has proprioceptors that encode velocity + length, length only, and force. These are group Ia muscle spindle afferents (*Eq. 6*), group II muscle spindle afferents (*Eq. 7*), and Golgi tendon organs (Ib, *Eq. 8*), respectively. Group Ia and II spindle afferents are subject to dynamic and static fusimotor modulation, respectively, controlled by the βMNs. βMNs are well described in amphibia (35) as are muscle spindles (36), which have also been described in the jaw muscles of fish (37). In-depth musculoskeletal system dynamics and receptor sensitivities and the rationale for βMNs can be found in our previous paper (27). Each limb has also a nondirectional cutaneous receptor (*Eq. 9*), which was modeled as a decaying representation of the net force applied onto the limb while pressing against the wall or the other limb. One additional feature of the cutaneous receptor was that the decay would plateau at 50% during steady-state net force, similar to a slowly adapting type I receptor (38)

(*6*) $$\text{Ia}=\frac{\left(1.5+{\text{log}}_{10}\left(A+0.1\right)\right)\text{*}V+A+\left(\text{II*}0.2\right)}{2}\mathrm{, (spindle Ia),}$$

(*7*) $$\text{II}=\frac{\left(L-0.2\right)\text{*}1.25+A}{2.0}\mathrm{, (spindle II),}$$

(*8*) Ib=L*A, (spindle Ib),

(*9*) $$C={\text{φ}}_{K}\left(F\text{*}0.5{\text{*φ}}_{\text{τ}}\left(\text{τ}\right),K_{C}\right)\mathrm{, (cutaneous),}$$

(*10*) $${\text{φ}}_{K}(x,\;K)=y_{t}=y_{t-1}\left(1-K\right)+x_{t}K\mathrm{, (Kalman filter).}$$

### Neuron Model

We used a nonspiking, linear summation neuron model with dynamic leak (39), which has a time-continuous, positive-only, output voltage signal (*P^+^*). Here, as well as in the companion paper (27), the neuron model was endowed with learning rules similar to those of a previous simulation of cuneate neurons (40), which is an unsupervised, Hebbian-inspired learning rule combined with a calcium co-variance learning rule (41, 42).

### Neuronal Compartment Model

The neuron model has a somatodendritic compartment (*Eq. 11*), which is electronically compact, and individual synaptic compartments, where each compartment has an independently regulated calcium concentration. The compartmentalization serves the purpose of controlling the learning (*Eqs. 12–16*) through the calcium covariance rule, so that the amount of correlation between the synaptic calcium activity and the somatodendritic calcium activity defines the net learning. The model is simplified such that the calcium concentration (as a dimensionless quantity) of the different compartments is equivalent to its voltage.

Rather than simulating the complex mechanics of membrane conductance explicitly, the effects of synaptic shunting and leak conductance were emulated using *Eq. 11*. Thus, the neuronal activation of the *i*th neuron depend on the positive activity from the *j*th neuron ($P_{j}^{+}$) modulated by the positive synaptic connection weight ($w_{ij}^{+}$) and a sign matrix where excitatory synapses are positive and inhibitory are negative (*S_ij_*). The shunting is represented by the sum of synaptic activation plus a resting leak (*k_i_*) in the denominator. The resting leak, *k_i_*, is twice the rolling sum (with a lower bound of 0.5) of synaptic activation and is intended to reflect the size of the neuron. As such it is only allowed to increase with time (after an initial stabilization phase has passed, which was set to 30,000 s). The summed synaptic activity in both the numerator and in the denominator are low-pass filtered using a Kalman filter as described in *Eq. 10*.

(*11*) $$P_{i}=\frac{\varphi_{K}\left(\sum P_{j}^{+}w_{ij}^{+}S_{ij},\;K_{A}\right)}{k_{i}+\varphi_{K}\left(\sum P_{j}^{+}w_{ij}^{+},\;K_{A}\right)},�(neuronal�activation).\mathrm{, (neuronal activation).}$$

### Synaptic Weights

The synaptic weights in our model are scalars with a functional range of 0 to +1. There is no information on fetal synaptic weights, and thus we made the assumption that synapses have randomized initial weights at the low end of the spectrum. In our model, the initial synaptic weights are random samples from a normal distribution (mean µ = 0.2, standard deviation σ = 0.16, synaptic weights below 0.001 were reseeded). The Ia to βMN synaptic weights were preset to the strong homonymous pattern that emerged in our previous simulation (27) and were not allowed to learn further. The model included 16 excitatory and 16 inhibitory interneurons whose postsynaptic effects had a fixed sign according to a static matrix (*S*).

### Learning Rule

During the course of a simulation, the synaptic weights were continuously adjusted according to the learning rule, which captures the temporal correlation between activity of individual synapses and the somatodendritic compartment. This emulates the calcium co-variance rule for learning (41, 42), which enables both continuous Hebbian LTP and LTD using a slight variation to Oja’s rule (43) (*Eq. 12*). Notably, all excitatory and all inhibitory synapses are learned continually throughout simulations. The scalar learning signal (*Eq. 13*, range −1 to +1) allows for a graded update for each individual synapse. For learning in excitatory synapses, the learning signal was slightly high-pass filtered to emulate the faster supralinear calcium signal that is generated under excitatory synapses, whereas for inhibitory synapses the learning signal was instead low-pass filtered to reflect the slower kinetics of the calcium channels surrounding them (40). Depending on the sign of the learning signal, the compensation factor is updated accordingly (*Eq. 14*). Finally, a learning rate parameter scales the learning (*Eq. 15*)

(*12*) $$\Delta w_{ij}=l_{ij}\eta_{i}c_{ij}\mathrm{, (learning rule),}$$

(*13*) $$l_{ij}=\varphi_{K}(P_{j}^{+}w_{ij}\left(\varphi_{L}\left(P_{i}\right)-{\bar{P}}_{i}\right),\;K_{L})\mathrm{, (learning signal),}$$

(*14*) $$c_{ij}=\{\begin{array}{c} 1-w_{ij},\;l_{ij}\geq 0\\ {\text{w}}_{ij},\;l_{ij}<0 \end{array}\mathrm{, (compensation factor),}$$

(*15*) $$\eta_{i}={\bar{P}}_{i}^{4}\text{*}0.01\mathrm{, (learning rate),}$$

(*16*) $${\bar{P}}_{i}=\varphi_{K}(P_{i}^{+},\;K_{M})\mathrm{, (mean activity).}$$

### Muscle Activation

Each of the four Oropod muscles is controlled by a single β-motoneuron (βMN) (Fig. 1, *B* and *C*). However, muscles are not commonly controlled by a single MN, but rather a motor pool of hundreds of MNs. These motor pools enable gradual activation of the muscle by cumulative recruitment of motor units. Based on this observation we reduced the motor pool of each muscle into a single βMN that, as defined earlier, had a scalar output (*P^+^*, positive part of *Eq. 11*). The strength of the muscles was at 100% as defined in our previous publication, i.e., we defined full muscle strength to be when the organism could move one limb from one extreme position (full flexion/extension) to the other in roughly 2 s. Control of each βMN is similar to that of the companion paper (27) with one minor difference regarding their inhibitory control. The previous model contained only the excitatory Ia-βMN synapses, so we had to introduce a small hyperpolarizing bias to each βMN to prevent the muscles from being continuously active. This bias was not needed during the simulations described here because we had inhibitory neurons (Fig. 1*C*) that could cause muscle relaxation and the muscle activation (*A*) could therefore be defined as a direct reflection of the βMNs activity (*P^+^*; *Eq. 17*).

(*17*) $$A=P^{+}\mathrm{, (muscle activation).}$$

### Autonomous Activity Pattern Generator

A crux in any organism, or model thereof, is how motor activity initially arises and how it is subsequently molded into useful behavior. A common resolution of this issue in the mature spinal cord is the notion of a central pattern generator (CPG) (44). However, the CPG is itself often considered a mature and informed machine capable of producing functionally synergistic contractions of muscles in the musculoskeletal system to which it is attached. How the CPG reaches such a mature and informed state is unclear.

Mechanistically, coordinated pacemaker potentials in early developmental stages by means of voltage-gated calcium channel expression (45, 46) and gap junctions between βMNs (47, 48) have been well described. We tried to capture this type of behavior in a twitch activity pattern generator (APG) that produced randomized, variable, and overlapping twitches of activity. The APG was implemented as an independent excitatory synapse on each βMN set to the maximally allowed synaptic weight (1.0), which was nonadaptable. One advantage to this implementation was that as all the other types of synaptic inputs to the βMN gradually increased, and the activity of the spinal interneurons also gradually increased, the synaptic shunting effect (see *Eq. 11*) would continuously reduce the relative impact of the APG and thereby gradually increase the relative impact of the interneuronal synaptic inputs to the MNs.

Each twitch APG pacemaker potential has four parameters: amplitude, duration, rise time, and probability of activation. Each parameter was varied to avoid biasing the model toward any particular solution. Amplitude was assigned a random value between 0 and 1. Duration was assigned a value between 50 and 500 ms. Different rise times were controlled by applying a one-dimensional Kalman filter to the potential with a randomly assigned gain between 0.5 and 0.8. All values mentioned earlier were assigned from a uniform distribution for each new twitch (Fig. 1*D*). Most importantly, the probability of activation governed the frequency and potential overlap of muscle twitches. A probability set to 0.5 would result in an equal presentation of all 16 combinations available [4 muscles, 2 categorical states (ON/OFF), $2^{4}=16$ combinations]. A lower probability would increase the frequency of states with no activity, and a higher probability would increase the frequency of states with cocontraction. For the simulations presented in this paper, we assigned the probability of activation to 0.5. A deeper investigation of effect of APG settings was made in the companion paper (27). A sample of the twitch pattern used for training in this paper is shown in Supplemental Fig. S1 (see https://doi.org/10.6084/m9.figshare.19161905). These twitches did not generate sufficient force on the walls to shift the position of the Oropod body.

To test the behavior of the spinal circuitry during more organized limb movements, we used a phasic APG that generated strong, alternating patterns of muscle activity as illustrated in Fig. 7 and discussed in the associated text. The Hebbian plasticity was turned off during these tests.

### Simulation

The nervous systems of animals have a closed loop of continuous sensorimotor activity. This means that the mechanical behavior in conjunction with the physical properties of the external world affect the sensory feedback projected into the nervous system, which is combined with internal activity that in turn controls end-effector organs (such as muscles). We emulated this loop with our artificial musculoskeletal system (Fig. 1, *A* and *B*), our artificial nervous system (Fig. 1*C*), and an artificial physical world as follows:

1. The state of the Oropod musculoskeletal system and its interaction with the surrounding physical world was continuously updated by the physics engine (pymunk, http://www.pymunk.org/) using an integration time step of 10 ms.
2. The contractile force generated by each muscle in the musculoskeletal system was updated according to its current length and the output from each corresponding βMN, thus updating the state of the musculoskeletal system.
3. The time evolving activity of the proprioceptive sensors, APG twitches, interneuronal and βMN activity was integrated as described earlier, with respect to output and adjustment of synaptic weights accordingly, resulting in an updated output from each βMN.

The transmission of data from a source to a neuron goes through a synaptic connection. The synaptic connectivity of the simulated neuronal system can be visualized as a matrix with neurons as rows and incoming synaptic connections as columns; an example can be seen in Fig. 3*A*. Each of the four motoneurons receives sensor data from the Ia sensor in each of the four muscles. The four Ia synapses were preset at fixed values that emerged during a similar self-organizing process described in the companion paper (27). Here, we added synaptic connections from 16 excitatory and 16 inhibitory interneurons, and finally one APG input. Each motoneuron thus has in total 37 synaptic connections (36 plus the special APG synapse), of which the 32 interneuronal synapses were initiated with random seed weights and subsequently subjected to Hebbian learning. The interneurons received input from all of the Ia, Ib, and II afferents from each muscle plus one cutaneous input from each limb, resulting in 14 primary afferent synaptic connections, plus 31 interneuronal connections (one less than to the motoneurons, because an interneuron did not synapse onto itself) resulting in a total of 45 synapses each.

We saved the state of the system every 500 s throughout our simulations. This allowed us to load any saved state and run analysis on that particular state. Each simulation ran for 400,000 s (4.6 days of simulated development), which was a duration that allowed the synaptic weights to adapt to a stable configuration and demonstrated that the state was stable over time.

We could load a state into the loop described earlier and let it perform a specified set of movements. This was achieved by replacing the twitch APG with the phasic APG that produced a fixed set of muscle combinations (defining which βMNs to activate with specific amplitudes and durations; Fig. 1*D*). This was used to demonstrate how the evolving spinal circuitry could modulate the execution of simple behaviors. These sessions with the phasic, testing APG were never a part of the training simulations.

### Dimensionality Analysis Using Principal Component Analysis

Both the distribution of the synaptic weights, or the “synaptic landscape,” of each neuron and the distribution of the activity across that population of neurons constitute high-dimensional hyperspaces whose predominant states can be extracted by principal component analysis (PCA). PCA finds an ordered set of orthogonal dimensions (principal components, PCs) along which the variance is the greatest. The first PC dimension accounts for the greatest amount of variance of the data. To position each datapoint in the “PC-space,” the coefficient of each PC is calculated, i.e., how far along that dimension is the datapoint located.

The weight of each synapse onto a neuron can be seen as a dimension along which the neuron is located. Each βMN has 36 synapses (not counting the APG) and each interneuron has 45 synapses. This means that each βMN has a 36-dimensional space within which it is located according to the distribution of the weights across those 36 synapses; interneurons have a location in a 45-dimensional space. To compare to what extent the dimensionality of each space was necessary to describe the neuronal connectivity, we applied PCA both to reduce the dimensionality and also to quantify how much each new dimension (PC) accounted for the total variability within the space. This measure was important to determine the outcome of the learning in terms of the diversification of the connectivity of the neurons. A high diversity of the learned connectivity would thus require a high number of PCs to be accounted for, whereas if neurons instead were highly similar to each other in terms of their connectivity, they could be fully accounted for with just a couple of PCs. Because we wanted to compare the dimensionality of all neurons but interneurons did not make synapses onto themselves, we replaced the nonexistent synapses (the magenta cells in Fig. 3*A*) with a synaptic weight of 1.0 because a neuron would be 100% correlated to itself.

We also used PCA in an entirely different analysis that was instead focusing on the distributions of activity across all 32 interneurons (16 excitatory, 16 inhibitory; see Fig. 1*C*) during various phases of activation (Fig. 7). In this analysis, we performed the PCA on the population-wide activity distribution on every single timestep across all movements. These results are visualized using only the first 3 PCs/dimensions (Fig. 7, *D*–*O*). However, the measure of the total distance traveled in this activity distribution “space,” we used all the number of PCs/dimensions that were required to explained at least 95% of the total variance of the data (Fig. 7*P*).

## RESULTS

Our model creature with simple musculoskeletal mechanics, biologically derived sensor tunings, and adaptive neural network was used to test the hypothesis that initially undifferentiated sets of excitatory and inhibitory interneurons can result in the formation of functional circuits through the process of Hebbian learning. We performed 10 simulations with different initial randomizations of synaptic weights (Fig. 1*E*, *top* for example simulation) and random seed value was used for the generation of twitch APG activity. They all converged to stable states exhibiting differential distributions without excessive degrees of saturated or desaturated synapses [Fig. 1*E*, *bottom*; compare Supplemental Fig. S2 (see https://doi.org/10.6084/m9.figshare.19161821) for the remaining nine simulations].

### Evolution of Connectivity during Kinematically Stable Behaviors

**Figure 2.**
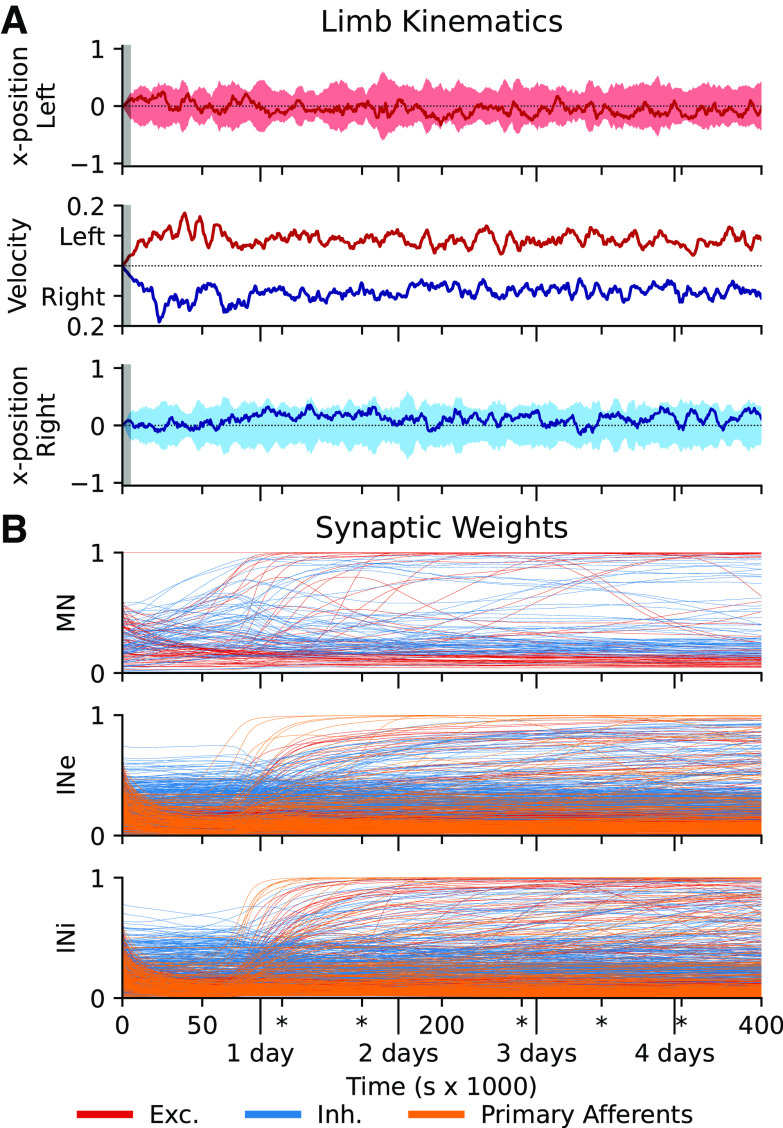
Development during the training cycle of the example simulation. *A*: limb kinematics during the training cycle, sampled every 500 s. *Top* and *bottom*: *x*-position relative to the center of limb travel (left limb: *top*; right limb: *bottom*) as a rolling mean (dark line) and range (lighter shading) over 5,000 s (initial zero-padding indicated by gray background). The rolling mean maximal velocity for each limb is shown in the *middle*, positive upward for the left limb and positive downward for the right limb. Note the slight increase in velocity and range of motion during the initial phase of the training but with no mean deviation from the neutral position. *B*: synaptic weight development for each neuron type during the example simulation. Each input source (excitatory interneuron, inhibitory interneuron, and primary afferent) is shown with separate color code. After roughly a days’ worth of experience, the synaptic weights of the network quickly stabilize, with only slow and sparse changes in the later stages. INe, excitatory interneurons; INi, inhibitory interneurons; MN, motoneuron.

Whereas the limb positions and limb speeds remained within the same space during the entire training (Fig. 2*A*), reflecting the strong influence of the APG on the movements, the synaptic weights of all connections of the network tended to change dramatically and in different ways (Fig. 2*B*). First, there was a loss of some of the seed weights, primarily excitatory, which is reasonable given that at least some of the randomized weights would not be functionally relevant for the system. Then there was a gradual gain of weights from the interneurons to the βMNs (during *day 0*, Fig. 2*B*, *top*). In conjunction with this, the interneurons gradually started to acquire more specific primary sensory afferent inputs, which was in turn followed by the formation of connections between the interneurons (around *day 1*). This is of particular functional importance because a functionally relevant connectivity between the interneurons could only be formed after the interneurons had acquired a mature sensory input and a coherent influence on the muscle activation that generates such sensory feedback. The sensory input is a more dynamic signal and therefore is more easily learned with these learning rules. The maturation of sensory input would gradually make the output of the spinal interneurons more dynamic, which would eventually make it possible to form meaningful connectivity patterns among the interneurons themselves. The rapid general increase in synaptic strength gradually leveled off during *day 1*–*2*, but many individual synapses of all types underwent pronounced increases or decreases during the subsequent consolidation period. This is reasonable given that the network during the training would be expected to find temporary solutions that would subsequently need to be superseded by even more appropriate solutions. This hypothesis is supported by the higher degree of late synaptic adjustments in the IN-βMN synapses, indicating that a slight adaptation within the interneuronal population informs a greater diversity that is then reflected as a distinct change of the efferent synaptic weights. The implications of this are described in *Diverse and Heterogenic Synaptic Landscape* with reference to Fig. 4. Note that we used no guidance or “critical time periods” for these plasticity processes or phases.

**Figure 3.**
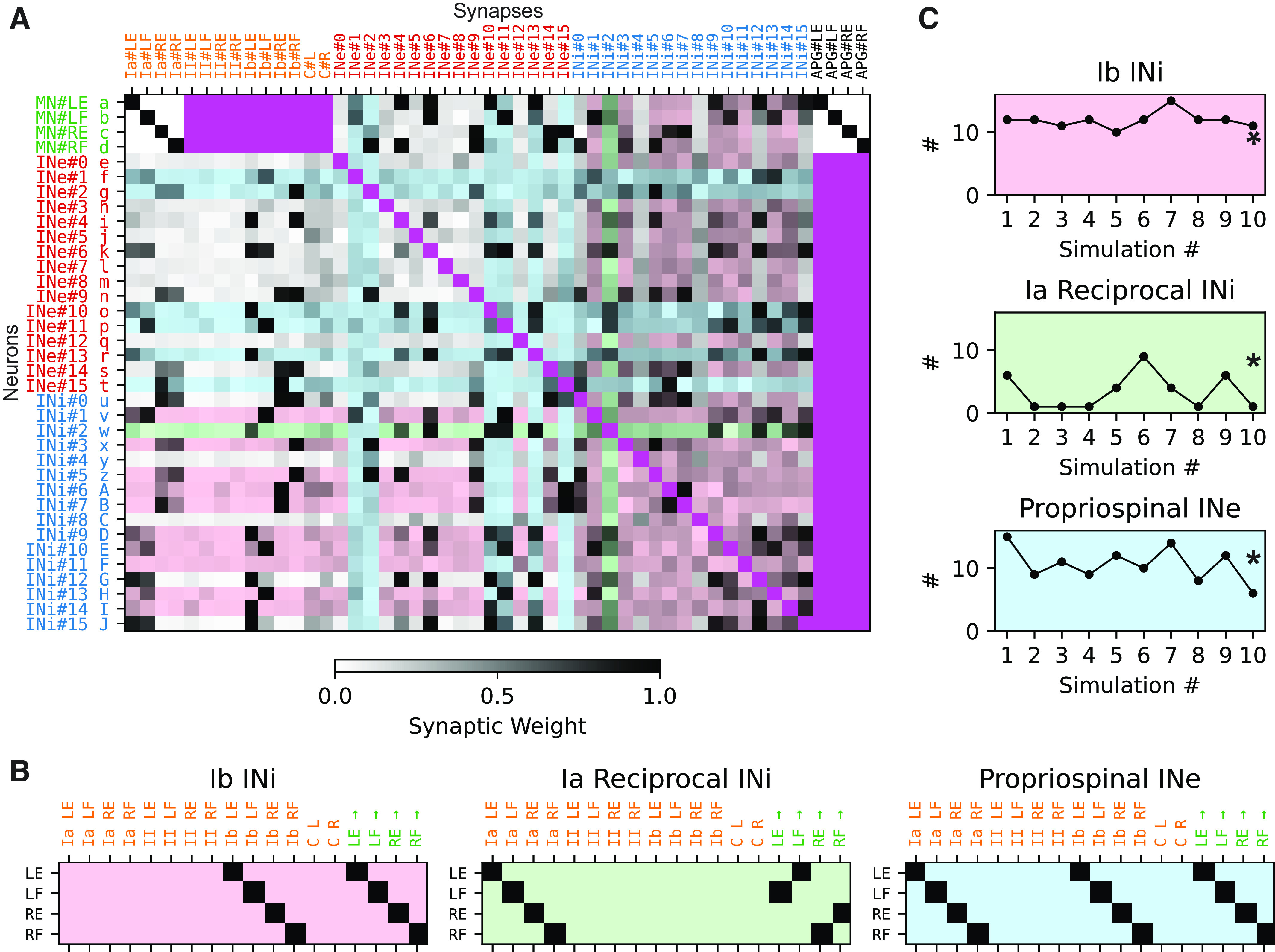
Connectivity matrix of the Oropod spinal cord following training. *A*: the connectivity matrix of a full spinal cord at the end of the example simulation (400,000 s). Each row indicates the synaptic input to a neuron in the neuronal network. Each row has a unique letter label a–J, used for identification in Figs. 4 and 5. Each column describes the output of a source of activity in the neuronal network. Each synaptic weight is indicated by gray scale. Archetypical interneurons are indicated by shading with the pastel color codes shown in *B* (both input/row and output/column). Cells that do not represent a synapse are colored a saturated magenta. *B*: three archetypical interneuron classes (from the literature): Ib inhibitory interneuron, Ia reciprocal inhibitory interneuron, and propriospinal excitatory interneuron, with background color key. Each row describes the connectivity of one instance of the archetype. The first 14 columns describe the prescribed synaptic input pattern, and the last four the synaptic output pattern. Thus, the archetypical Ib inhibitory interneuron for the left extensor (LE) receives input from the Ib sensor of the LE muscle and projects to the β motoneurons (βMNs) that controls the LE. *C*: prevalence of the three archetypical neuron classes (*y*-axis) across the 10 different training sessions (*x*-axis). The example training session is indicated with a star in each plot. INe, excitatory interneurons; INi, inhibitory interneurons; LE, left extensor; LF, left flexor; RE, right extensor; RF, right flexor.

**Figure 4.**
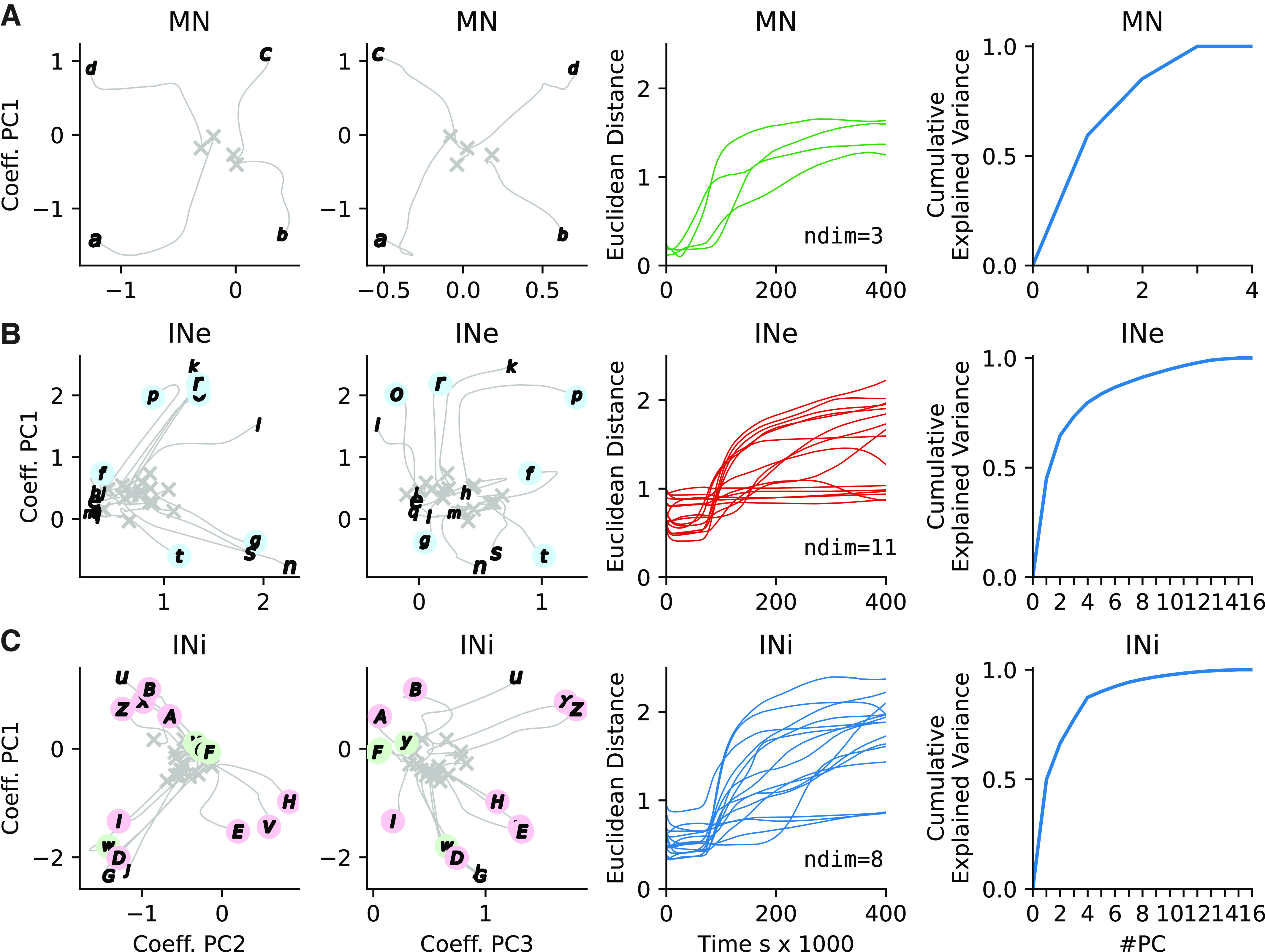
Evolution of synaptic input weights across the neuron types during the example training session. *A*: For the 4 β motoneurons (MNs) (*a*–*d*), the evolution of the patterns of synaptic input connectivity from the 32 spinal interneurons, quantified by their coefficients principal component (PC) 1 vs. 2 of the synaptic patterns. First column, gray Xs indicate the originally seeded weights with gray lines extending to the end weights labeled as defined in Fig. 3*B.* Second column is identical to the first except that the visualized PCs are 1 vs. 3. Third column plots the Euclidean distance of the synaptic inputs from initial mean seed value to fully trained. The distance is calculated in a *n*-dimensional PC-space where *n* is the number of PCs needed to account for 95% of the variance (labeled as ndim). Fourth column plots the cumulative explained variance of the synaptic input patterns for each added PC. *B*: similar display for the 16 excitatory interneurons (INe) (*e*–*t*), but including also the weights of the primary sensory afferent synapses onto the INe. *C*: similar display for the inhibitory interneurons (INi) (*x*–*J*). In *B* and *C*, circular markers in pastel colors indicate interneurons with archetypical connectivity as defined in Fig. 3.

### Emerging Archetypical Interneuronal Connectivity

The connectivity matrix of a full spinal cord after a completed training session is illustrated in Fig. 3*A.* A striking feature is the overall diversity of the connectivity for both primary afferents to interneurons and interneurons to interneurons. Such complexity does not lend itself to simple visualization of patterns. As a first approximation, we utilized the definitions in the neurophysiological literature of what we here refer to as “archetypical” interneurons (49, 50). We developed an objective method to identify interneurons whose connectivity conformed to the archetypical connectivity patterns of Ib inhibitory interneurons (mediating Ib disynaptic inhibition to the homonymous muscle), Ia reciprocal inhibitory interneurons (mediating Ia disynaptic inhibition to the antagonistic muscle), and excitatory propriospinal neurons (convergent Ia and Ib input to the homonymous muscle) [color coded in Fig. 3, *A*–*C*; see Supplemental Fig. S3 (see https://doi.org/10.6084/m9.figshare.19161908) for the remaining nine connectivity matrices obtained from simulations with different seed weights]. We used these input/output archetypes to create the pure connectivity templates for all four muscles shown in Fig. 3*B*. We calculated the Euclidean distance (L2-norm) from the archetypical connectivity to the actual connectivity of each interneuron in the connectivity matrix in Fig. 3*A*. An interneuron was classified as adhering to an archetype if and only if one distance to an archetype combination was below the mean population distance minus one standard deviation. For example, if an inhibitory neuron had 100% synaptic weight of the Ib primary afferent (PA) from the left extensor, and 0% weight from all other PAs, and also had 100% synaptic weight in the efferent synapse unto the left extensor βMN together with 0% weight in the other βMNs, then that neuron would have zero distance to the Ib inhibitory left extensor interneuronal archetype (Fig. 3*B*, *left*, *top row*). We did not set a lower/upper bound for any of the particular synaptic connections, instead focusing on the overall distance between the archetype and the actual. This had the effect that some neurons with weak, but accurate, connectivity still could be classified as belonging to a particular class (see Fig. 3*A*, INi#2 classified as Ia reciprocal as an example).

The relative prevalence of archetypical connectivity patterns varied somewhat across the 10 different, initially randomized simulations, but overall remained relatively constant (Fig. 3*C*). The analysis was made separately for each archetype, meaning that the same neuron could in theory be classified as belonging to multiple archetypes. Such multiclassifications occurred in a mean of 3.3 neurons (SD = 2.65) across the simulations (*n* = 10). However, such multiclassification is to our knowledge not in conflict with the descriptions in the literature, as discussed later.

### Diverse and Heterogenic Synaptic Landscape

Our next analysis focused on the diversity of the input connectivity for each neuron of the Oropod spinal cord network (Fig. 4). To compare the input connectivity to the different neurons of the network, we performed a PCA of the distribution of synaptic weights for the specific inputs across all the neurons of each given type. We further quantified these synaptic weight distribution patterns across different training phases and followed their gradual evolution.

For the βMNs, their synaptic weight landscapes almost immediately started to diverge in unique directions, and at the end of the training they had highly disparate positions in the PC space (Fig. 4*A*, *first and second column from the left*). Across all simulations, the first two PCs explained a grand mean of 83.9% (SD = 3.9%, *n* = 10) of the variance of their input connectivity. This indicates that each βMN came to receive inputs from unique combinations of spinal interneurons. As we had only one βMN per muscle, this would mean that each βMN received interneuronal inputs that were associated with the activation of that muscle, i.e., information that at some point had proven relevant for that muscle activity. In terms of how that specificity evolved over time, we also measured the Euclidean distance traveled (within the PC space of the synaptic input patterns; Fig. 4*A**, third column from the left*). Maximal development for the βMNs in this respect occurred between 25,000 and 125,000 s. We also found that for the four βMNs, three PCs were required to account for a large proportion (>95% of the variance) of the synaptic input patterns observed (Fig. 4*A*, *fourth column from the left*), again compatible with the observation that each βMN had essentially unique synaptic input patterns from the spinal interneurons. Across all 10 simulations, three PCs were required to explain at least 95% of the variance (SD = 0.0, *n* = 10).

For the excitatory spinal interneurons, the picture was more complex (Fig. 4*B*), with more disparate input connectivities, but also some neurons for which the synaptic weight distribution patterns did not evolve dramatically from their initial random seed weights. Across all the simulations, the first two PCs accounted for a mean of only 67.3% of the variance (SD = 7.8%, *n* = 10) for the excitatory interneurons. For the inhibitory interneurons (Fig. 4*C*), there was a tendency to form a few main end-point groups. The mean explained variance of the two first PCs for the inhibitory interneurons was 68.7% (SD = 6.2%, *n* = 10). Importantly, however, the number of PCs required to explain 95% of the variance of the synaptic input patterns were for both INe:s and INi:s much higher than the two PCs used for the illustrations of the evolution within the PC space (Fig. 4, *B* and *C*, *fourth column*; INe mean = 9.2, SD = 1.83, *n* = 10 simulations; INi mean = 6.7, SD = 1.49, *n* = 10 simulations). In other words, the diversity of synaptic input distribution patterns among the spinal interneurons was much more diverse than can be appreciated from these two-dimensional (2-D) plots (first and second columns in Fig. 4, *B* and *C*). It can also be noted that the most dramatic phase of evolution of the synaptic input distribution patterns for the interneurons occurred between 70,000 and 175,000 s. Curiously, the distance metric for many neurons continued to increase throughout the subsequent consolidation period. The implications of this for functional behaviors are discussed later (see Fig. 7).

### Ontogenetic Imprinting in the Interneuron Subnetwork

**Figure 5.**
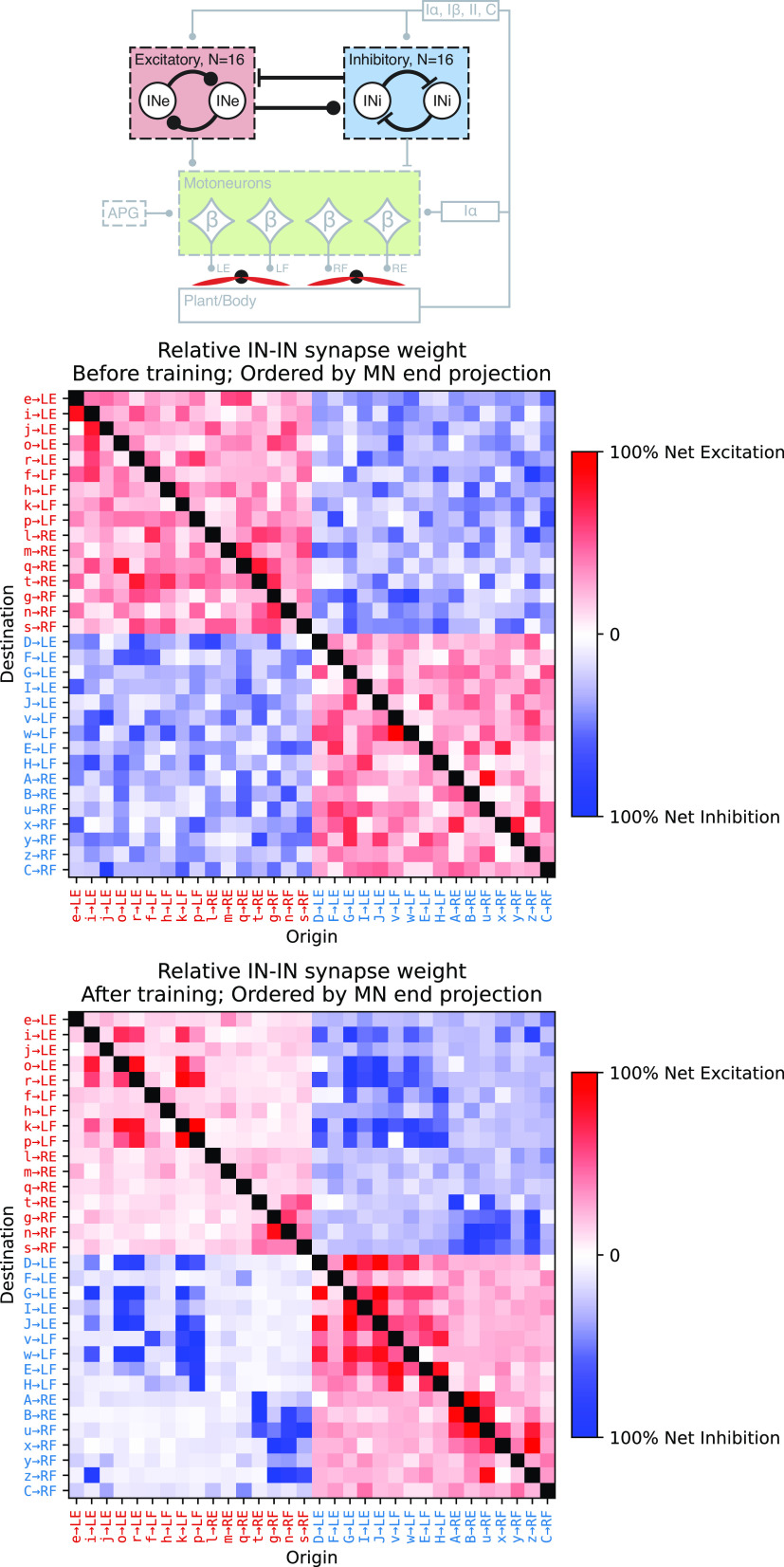
Training-induced connectivity patterns between interneurons as specified by the illustration at *top*. *Middle* and *bottom*: the synaptic weights before and after the example training session. Each row describes the synaptic input to an interneuron and each column describes the output. The excitatory interneurons (INe, red labels) and inhibitory interneurons (INi, blue labels) are separated and regrouped according to the β motoneuron (βMN) to which they have the strongest synaptic projection [left to right order of left extensor (LE), left flexor (LF), right extensor (RE), right flexor (RF)]. The row and column labeling of the interneurons consists of the unique letter label from Fig. 3 followed by the identity of the βMN that receives the strongest projection from that interneuron. The black cells are nonsynapses.

Related to the issue of connectivity patterns, we separately investigated the patterns of connectivity between the spinal interneurons (Fig. 5). The matrices illustrate the connectivity pattern between interneurons before (Fig. 5, *middle*) and after training (Fig. 5, *bottom*) for the example training session. The excitatory and inhibitory interneurons were sorted on the basis of the βMN (limb, flexor/extensor) to which they had the strongest output at the end of training. The main observable effect was that interneurons innervating βMNs of the same limb (i.e., regardless of whether it was the flexor or extensor) had a high tendency to form strong connections to each other regardless of whether they were excitatory or inhibitory. There was a further tendency for interneurons of both classes to form the strongest connections to other interneurons with the same βMN target. This was most probably driven by an acquired specialization with respect to limb and sensory modality supported by the primary sensory afferents.

**Figure 6.**
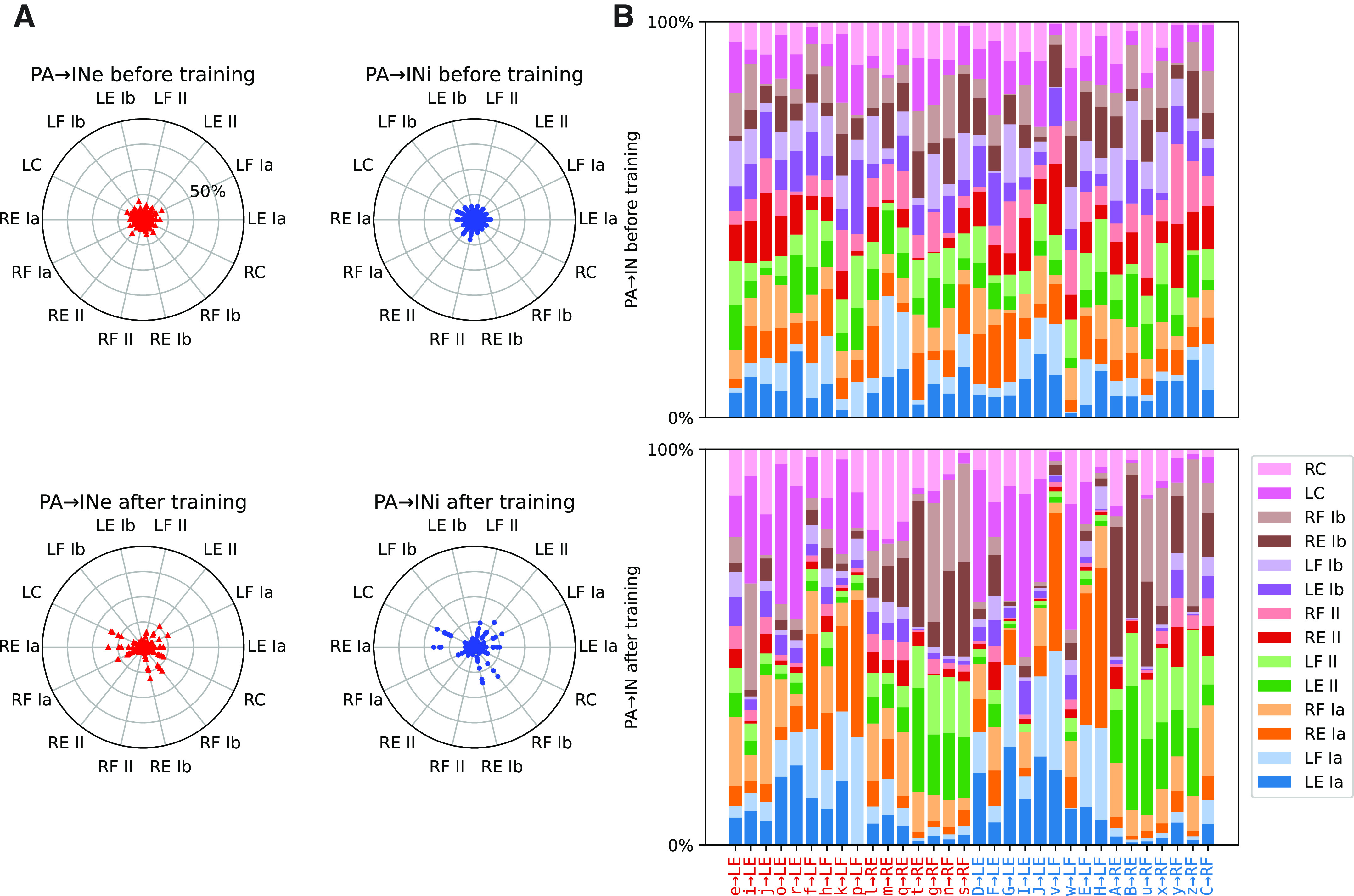
Training-induced development of primary afferent input connectivity in the interneurons in the example simulation. State before training is shown in the top row, whereas the bottom row shows the state after 400,000 s of training. *A*: the synaptic weight for each primary afferent relative to the total synaptic weight of the primary afferents. Radial plots for excitatory interneurons (INe, *left*) and inhibitory interneurons (INi, *right*) before (*top*) and after (*bottom*) training. Similar pairs of proprioceptive sensors are on opposite sides of the circle. The initial homogenous spread is replaced with a heterogeneous specialization with training. *B*: stacked bar chart of the relative primary afferent synaptic weight distribution before (*top*) and after (*bottom*) training. The interneurons have the same order as in Fig. 5., i.e., ordered according to their strongest efferent projection onto the β motoneurons (βMNs). Once again it can be seen that the initially random relative strengths give way to specificity for both sensory modality and limb origin. LE, left extensor; LF, left flexor; PA, primary afferent; RE, right extensor; RF, right flexor.

We next grouped the interneurons according to their acquired muscle targets to explore if the training resulted in correlated patterns of input from primary afferents (Fig. 6). Most interneurons replaced their initially weak and randomized primary afferent inputs with an increased specialization with respect to limb and sensory modality, thereby becoming more distinctive in their connectivity patterns. This can also be seen in Fig. 6, where the end weights of the primary afferent synapses have a more heterogeneous distribution compared with the seed weights (Fig. 6). Note that the cutaneous inputs, which were generally weak even at the end of adaptive development (Fig. 3), started to have relatively strong inputs (compared with other inputs) to a subset of both excitatory and inhibitory interneurons. The cutaneous inputs were sometimes represented unilaterally, as might arise from contact with the walls, and sometimes bilaterally, as might arise from contact between the two limbs.

### Emerging Functional and Cooperative Spinal Circuitry

**Figure 7.**
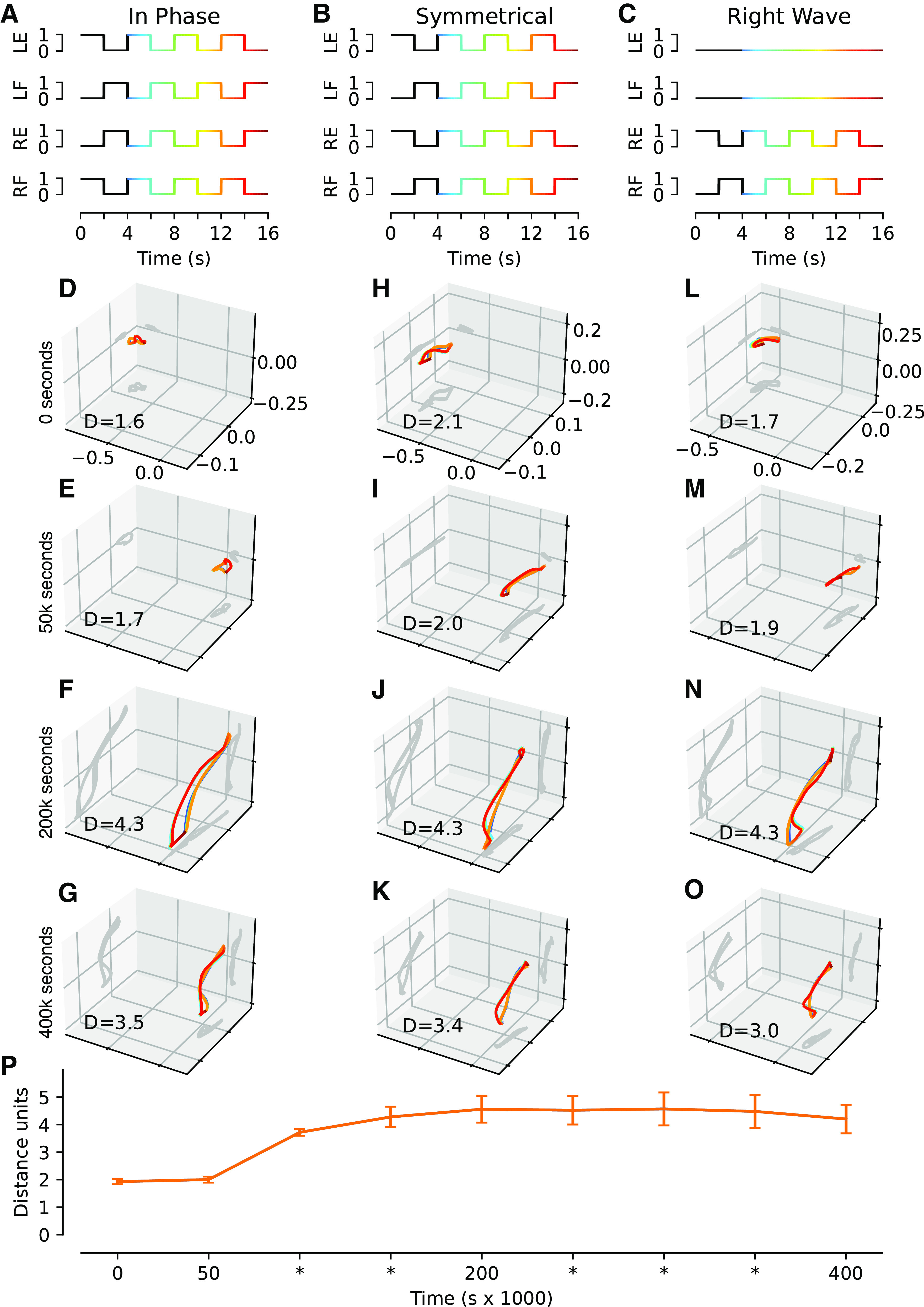
Neuronal cooperativity as assessed by principal component analysis (PCA) of the activity distribution across the interneuronal population for three different types of movements. We exercised the Oropod with predefined, phasic movements at different moments in training. The activation profiles for in phase (*A*), symmetrical (*B*), and right wave (*C*) movement show the activity that was injected into the activity pattern generator (APG) synapses instead of random twitches. The color gradient indicates the temporal phase of the specific movement. Each activation profile consists of three cycles of the core movement (4 s of duration), preceded by a cycle to initiate the system into a steady state (black line color in *A*–*C*). These phasic movements were not part of the training. *D*–*O*: the activity of the population of interneurons in principal components (PC) space during each of the activation profiles (one column for each movement), at four timepoints during the example training. In these plots, the interneuronal activity is reduced from 32 dimensions (one per interneuron) to the first three PCs; the corresponding 2-D projections are shown as dark gray shadows cast on their respective plane. The distance traveled is indicated for each plot. *P*: grand mean distance traveled in *n*-dimensional PC-space for all simulations across nine timepoints during training, where PC-space includes all dimensions required to account for 95% of the variance; error bars indicate SE range for all 10 simulations. LE, left extensor; LF, left flexor; RE, right extensor; RF, right flexor.

We next aimed to quantify if the outcome of the learning after a completed training session could have any measurable beneficial effects on system behavior (Fig. 7). Because the training session consisted of “motor babbling” from our twitch APG, i.e., erratic and inconsistent movements mixed in random order (Supplementary Fig. S1), it could not be expected that our system would learn to become better at generating any specific movement. In fact, in an untrained, randomly connected, fully recurrent network containing excitatory and inhibitory interneurons in equal proportions, it is even likely that the neurons would tend to cancel each other out rather than cooperate. This effect would be expected to increase with the number of neurons in the network, reflecting the central limit theorem. This noncooperativity could manifest, for example, when a primary afferent synaptic drive of an excitatory neuron is cancelled out by simultaneous activity in an inhibitory interneuron that also connects to the excitatory neuron. Hence, the untrained system would be expected to have poor cooperativity, in the sense that its neurons would be at risk of providing internally conflicting signals. This would lead to inefficient input-output relationships of the network. In terms of network, this would translate to shallow local minima and therefore unstable network behavior during the course of a longer movement trajectory.

A possible beneficial outcome of network training in a closed loop system is that the network learns to coordinate the activity of its neurons in utilitarian terms, and that it can find different network-wide cooperative modes across a wide variety of movement trajectories or movement patterns. To test this, we used a coherent (phasic) APG setting that was different from the twitch APG used for the training and therefore a completely new experience for the Oropod (Fig. 7). The measure of neuronal cooperativity that we used was designed to quantify the distance traveled in activity space for the population of spinal interneurons during such movements. If a neuron is highly active during one specific phase of a movement trajectory but essentially inactive at another phase, then it has made a long travel in its activity space. If the neuron has many such ups and downs during the full duration of the movement trajectory, then the distance it traveled in its activity space has become even longer. However, for the whole population of interneurons to display high cooperativity, it requires that the neurons have activity patterns that are different from each other across the duration of the movement trajectory. One reason for this is that the inhibitory neurons, which inhibited other neurons with strong synaptic weights after training (Fig. 3*A*), would tend to cancel out the activity of the excitatory neurons if the time profiles of their respective activities were overlapping. This would render the network inefficient because most of the neural activity would consist of the inhibitory neurons cancelling out the excitatory synaptic inputs to the excitatory neurons. Instead, well-timed neural activity patterns where different neurons contribute to the drive of the muscle activation across different phases of the movement trajectory would be a more efficient use of the energy required to generate neural activity. To obtain a proper measure of neuronal cooperativity, we used PCA to quantify the diversity of activity distributions across the neuron population across the different phases of the movement trajectory.

We performed the PCA of the activity distribution at several timepoints during training, when the training was temporarily stopped and the Oropod was exercised through the predefined series of test movements (Fig. 7, *D*–*O*). Within the resulting PC space, we compared the distance traveled by the network system. To explain 95% of the variance of the interneuronal population activity across all timepoints and movements, a mean of 5.1 PCs (SD = 0.3, *n* = 10) were required. Note that in the three-dimensional (3-D) plots visualizing the trajectories, only the first three PCs [which accounted for 84.4% (SD = 1.6%, *n* = 10) of the variance] are shown.

To verify that the cooperativity pattern was movement specific rather than fixed, we visualized three different types of movement patterns as generated by three different, phasic APGs. As shown in Fig. 7, *D*, *H*, and *L*, before training the neuron population activity was constrained to a small part of the activity space. After training, different movement patterns caused the same network to utilize different parts of its activity distribution space and to travel longer distances in this space (Fig. 7, *G*, *K*, and *O*). Interestingly, the first visible change was a dramatic movement of the “center of gravity” of the trajectory rather than a substantial change in the configuration of the trajectory (compare Fig. 7, *D* and *E*, *H* and *I*, and *L* and *M*). The quantified distance traveled in the full PC space that accounted for 95% of the variance is summarized in Fig. 7*P*, which indicates that maximal neuronal cooperativity was already reached within 200,000 s, which would then correspond to the time when the training had its peak effect on the function of the spinal cord circuitry. In fact, at the last time point of the training, the distance traveled instead declined, suggesting that the neuronal cooperativity actually became worse with exaggerated training. Interestingly, 200,000 s matched the timepoint when the greatest diversification of the neuron connectivity had been reached (Fig. 4). However, the continued increase in synaptic input pattern heterogeneity indicated by the analysis in Fig. 4 might suggest that the network here exhibited overtraining. Notably, though, if overtraining was the case, there was still a wide time window of training over which there was a maximal level of network functionality (Fig. 7*P*).

## DISCUSSION

We have demonstrated that a model spinal neuronal network, composed of excitatory and inhibitory spinal interneurons, which was connected to a model musculoskeletal system with biologically relevant muscle and sensor properties, can acquire a connectivity structure reminiscent of that observed in adult mammals based solely on Hebbian-style learning.

### Features of the Oropod Spinal Interneuron Network and Learning

Our model system was able to learn using straight-forward Hebbian learning, a form of learning that is supported by the presence of *N*-methyl-d-aspartate (NMDA) channels and classical LTP-like synaptic plasticity in spinal circuitry (51–54). Previous attempts to model aspects of spinal cord circuitry relied on anti-Hebbian (“anti-Oja”) learning (55, 56) for which there is no demonstrated physiological mechanism in the spinal cord. In addition, we assumed Hebbian learning also in inhibitory synapses, although plasticity in these synapses in early development remains to be demonstrated.

We used a previously published nonspiking neuron model (39) because the spike generation of neurons in the brain (57, 58) and the spinal cord (59) are known to be subject to some level of stochasticity, which is extremely difficult to capture accurately in models. Because output spike patterns of such neurons can be described as a probability density function of the underlying membrane potential (59, 60), each of our nonspiking neurons would emulate the stochastic behavior of small populations of neurons with similar time courses of membrane potential and without any intrinsic “local circuit” nonlinearities.

Importantly, our neurons had thresholds, so that their output could be silenced by synaptic inhibition. The inclusion of inhibitory neurons and their Hebbian plasticity further elevates the biological relevance of our model compared with previous models of adaptively learned spinal circuitry (55, 56). The actual threshold for each neuron was a dynamic component that depended on the activity of the network, which is more biological than typical artificial neural networks where the threshold/bias is fixed but neural activity can change instantaneously. Finally, our implementation of a dynamic leak and membrane time constant for synaptic integration (*Eq. 11*) prevented the network from falling into “parasitic oscillations” (39), which otherwise can occur in recurrent networks. This was an important element that helped our model system to maintain long-term stability. Further biological relevance comes from the fact that this neuron model emulates central features of conductance-based H-H neuron models (39).

### Limitations of the Oropod Musculoskeletal System and Spinal Cord

The design of the two-limbed, unidimensional Oropod is intended to provide the simplest model system in which to test principles of self-organization that lead to stable neural systems that would facilitate functional behaviors. Now that this has been achieved, further research should be possible to determine the effects of added musculoskeletal system complexity such as multiarticular limbs and muscles, dependence of muscle force on velocity, pools of variously sized MNs, pure fusimotor γMNs, etc.

Our network system did not include recurrent axon collaterals from motoneurons, so it was not possible to form the connectivity of Renshaw interneurons (inhibitory spinal interneurons excited by motoneuron axon collaterals). Notably, though, we observed the emergence of interneurons with modest connections to interneurons controlling the opposite limb (see Fig. 5), a potential analog to commissural interneurons (61, 62). Interneurons with commissural projections appear fairly early in embryological development (63). It is possible that the predefinition of such connectivity patterns by genetic surface markers or growth factors (64) makes them more prominent in vivo, absence of which might account for their sparsity in the learned Oropod spinal network. It is not clear, however, when the mature patterns of commissural connectivity responsible for alternating limb movements appear (65). The Oropod cannot generate body movements that would result in mechanical coupling between limbs until the muscles and activity patterns mature to generate forces high enough to overcome the stiction built into the Oropod body. If these mature patterns of commissural interneuron connectivity could arise from Hebbian learning, the Oropod model would therefore be able to generate them only in later stages of muscle and circuit development than studied here.

Our model system used a preprogrammed scheduling of connectivity learning only for the monosynaptic projections of Ia afferents to βMNs, which were then frozen at the end of this “critical period.” Other afferent types were prevented from making such monosynaptic connections. This restricted Hebbian learning depended on relatively strong fusimotor effects of βMNs compared with weak extrafusal muscle in early development, mandating a critical period after which further plasticity was blocked (27). The learning studied here occurred without any such constraint.

Despite the very limited mechanical complexity of the Oropod model system compared with limbed vertebrates, we found that interneuron types reminiscent of classical, neurophysiological “archetypical” connectivity patterns arose systematically across all our simulated learning sessions. In our model system, however, the patterns of connectivity of these interneurons appeared to be more in a continuum with many other interneurons. For example, from our single class of inhibitory interneurons there emerged some patterns of connectivity that were consistent with the Ib inhibitory interneurons, some that were consistent with reciprocal Ia inhibitory interneurons and some that were consistent with both (Fig. 3). Convergence of Ia and Ib input onto individual inhibitory interneurons has been inferred from intracellular recordings of IPSPs in motoneurons (66). A recent study using more sensitive voltage clamp methods failed to find a basis for separating such interneurons into discrete archetypes (67).

In addition to the continuum of distinct interneurons that emerged during our simulations, it was typical for a few interneurons to not end up in a differentiated state (e.g., INe e in Fig. 3*A*). The function of these weak interneurons is speculative. A possibility is that the information present in our system could be explained by fewer than 16 + 16 interneurons and the observed weak interneurons were excessive. This is a known feature of biological spinal development, which would justify apoptotic removal of these neurons after training (29). However, it is also possible that in our particular case these undifferentiated neurons will find a functional niche when descending commands through the corticospinal tract (CST) learn to generate more complex sensorimotor behaviors, a topic for future research. For example, cutaneous afferents have been reported to have short latency connections from human hand to leg motoneurons that may reflect learned functionality in later phases of development (68).

### The Relationship between Nature and Nurture

The fact that learning could potentially explain some major types of intraspinal interneuron connectivity described to date (of which the most common subsets are described in Fig. 3) does not exclude a role for genetically defined differentiation, guidance, and connectivity (69). Spatial gradients of cellular differentiation in the rostrocaudal and mediolateral axes appear to account for gradients in the density of sensory feedback from and to specific muscles (70, 71). Genetic differentiation and mutual recognition between cell-types presumably underlie general rules for connectivity such as the unique projection of recurrent collaterals from motoneurons onto inhibitory Renshaw interneurons (72, 73). The steadily increasing number of subtypes of neurons identified via transcriptomes (74, 75) may account for the many functions that were not part of our simulations such as ascending connectivity and autonomic regulation. Similarly, we did not attempt to model interneurons that constitute locomotor central pattern generators, which have been associated with distinctive transcriptomes (76) but whose circuit function remains contentious (77). Within those organizing rules of nature, however, there is room for muscle-by-muscle adaptation of interneuronal connectivity to reflect the mechanical dynamics of the musculoskeletal systems as determined by the experiences of nurture.

There are many good reasons for a network system involved in diverse functions such as the spinal cord to rely heavily on learning to define the functional details of its network structure. Learning can generate a diversity of interneuronal connectivities, rather than stereotyped, repetitive connectivities among subpopulations of interneurons, resulting in a more flexible and versatile substrate for neural control. If a particular type of connectivity turns out to be more useful than others, then it is likely to appear frequently and be identified experimentally whether it is the result of genetic prespecification or adaptive learning.

The identification of archetypes based on statistical trends in continuous distributions does not imply discrete mechanisms for generating them. Such archetypes can also be discerned in optimal regulators of musculoskeletal mechanics that include much richer patterns of connectivity (11). The historical association of simple archetypes with the simple connectivity expected for servocontrol (e.g., reciprocal length feedback and homonymous inhibitory force feedback) is misleading in two ways. It ignores the diversity of other connectivity identified experimentally in those neurons (9) and it ignores the complexity of the mechanical dynamics of individual muscles that act on one or more joints with multiple degrees of freedom (78) and that induce interaction torques on joints far from their anatomical origin and insertion (34, 79).

The role of learning should be to find as many different useful connectivity patterns as possible, thereby creating a diversity of interneuronal connectivities and activities (Figs. 4–7). Such functional diversity might then give rise to much of the transcriptional diversity observed in adult spinal interneurons, including a great proliferation of subtypes (75), at least some of which are known to be influenced by activity patterns (80, 81). If this is correct, it would imply that transcriptional markers of neuronal differentiation (82, 83) may be less determinative of spinal cord function than generally assumed. Many of these markers may reflect the effects of motor behavior, such as the associated neural adaptations that follows, rather than the cause.

A wider range of interneuronal connectivities can result in a network with a wider set of local minima solutions for a given behavior (16). Such a rich interneuronal population would allow a supraspinal controller to select different subsets of interneurons to perform different movements (Fig. 7). If the supraspinal controller learns to perform new behaviors as extensions of previously learned behaviors, such an interneuronal system generates solutions that can be interpolated to perform intermediate behaviors (17). That, in turn, gives rise to recurring patterns of muscle recruitment that have been claimed as evidence for the existence of hardwired synergies [for review, see Lee (84)]. Instead, the observed synergies may correspond simply to specific dynamic states induced in the spinal interneuronal network (85). The idiosyncratic complexity and stability of the emergent spinal circuitry further suggests that the brain discovers and remembers rather than computes these useful dynamic states as a result of orderly development of motor skills starting in infancy (13).

Our spinal networks rapidly found a potential solution space as seen in stable weights (Fig. 2*B*), heterogenic and wide distribution of these weights (Fig. 4), and cooperativity between interneurons (Fig. 7). Interestingly, the Euclidean distances for interneuronal connectivity continuously increased during extended training while the Euclidean distances for neural activity during phasic movements started to decline in the later stages of the simulations. This might reflect a form of over-training on the limited kinematics of random twitches. This might be avoided by the timely arrival of corticospinal projections capable of generating a richer set of limb movements, a topic for future research.

The results of adaptive learning in an integrated sensorimotor system such as the spinal cord will depend on the movements made, the biomechanical properties of the body, and the tuning of the sensors monitoring the biomechanical events associated with the movements, as well as the properties of the neurons and their learning rules. Early motor babbling would be an essential component of such learning, enabling the full potential movement space to be imprinted into a network that then provides many “good-enough” solutions that can be discovered by supraspinal controllers (12). Our APG consisted of randomly overlapping twitches of individual muscles, similar to those observed in early fetal development. In reality, all vertebrates appear to generate spontaneous movements that become more complex and diverse as fetal and perinatal development progresses (86–88). Natural pathologies and experimental interventions that deprive the fetal spinal cord of spontaneous motor activity result in impoverished circuit formation and behavioral deficits [for review, see Tahayori and Koceja (89)].

The simple Oropod model system has only 16 excitatory interneurons, but the possible (binary) combinations of activation are 65,536; if we add the 16 inhibitory interneurons, the possible combinations are 4,294,967,296. The corticospinal system affects voluntary behaviors largely by recruiting and derecruiting specific subsets of these interneurons. To know a priori which particular combinations are the ones that produce a particular movement would require a crystalline and precise knowledge that is beyond astonishing. A minuscule shift in any connection would risk the collapse of the entire system. Adaptation a posteriori instead can provide a robust substrate for open-ended learning of sensorimotor behaviors.

That evolution would tend to favor developmentally adaptive rather than genetically preordained mechanisms was first proposed by J. Mark Baldwin over a century ago (25, 26). Even if it were possible for simple organisms to function with genetically specified musculoskeletal architecture and neuronal circuitry, the evolution of new and more functional species would be favored by neural mechanisms that could adapt to variations of the musculoskeletal system and the environment in which it must function. The model presented here demonstrates that such adaptation is possible and stable using physiologically realistic components and processes.

## SUPPLEMENTAL DATA

10.6084/m9.figshare.19161905 Supplemental Fig. S1: https://doi.org/10.6084/m9.figshare.19161905;

10.6084/m9.figshare.19161821 Supplemental Fig. S2: https://doi.org/10.6084/m9.figshare.19161821;

10.6084/m9.figshare.19161908 Supplemental Fig. S3: https://doi.org/10.6084/m9.figshare.19161908.

## GRANTS

This work was supported by the European Union Grant FET 829186 ph-coding (Predictive Haptic COding Devices In Next Generation interfaces).

## DISCLOSURES

No conflicts of interest, financial or otherwise, are declared by the authors.

## AUTHOR CONTRIBUTIONS

J.M.D.E., G.E.L., and H.J. conceived and designed research; J.M.D.E. performed experiments; J.M.D.E., G.E.L., and H.J. analyzed data; J.M.D.E., G.E.L., and H.J. interpreted results of experiments; J.M.D.E., G.E.L., and H.J. prepared figures; J.M.D.E., G.E.L., and H.J. drafted manuscript; J.M.D.E., G.E.L., and H.J. edited and revised manuscript; J.M.D.E., G.E.L., and H.J. approved final version of manuscript.
